# Functional characterization of two defensin isoforms of the hard tick *Ixodes ricinus*

**DOI:** 10.1186/1756-3305-4-63

**Published:** 2011-04-19

**Authors:** Tereza Chrudimská, Jiřina Slaninová, Nataliia Rudenko, Daniel Růžek, Libor Grubhoffer

**Affiliations:** 1 University of South Bohemia, Faculty of Science, Branišovská 31, České Buděovice, Czech Republic; 2 Biology Centre v.v.i., ASCR, Institute of Parasitology, Branišovská 31, České Buděovice, Czech Republic; 3 Institute of Organic Chemistry and Biochemistry, ASCR, Flemingovo square 2, Prague, Czech Republic

## Abstract

**Background:**

The immune system of ticks is stimulated to produce many pharmacologically active molecules during feeding and especially during pathogen invasion. The family of cationic peptides - defensins - represents a specific group of antimicrobial compounds with six conserved cysteine residues in a molecule.

**Results:**

Two isoforms of the defensin gene *(def1 *and *def2*) were identified in the European tick *Ixodes ricinus*. Expression of both genes was induced in different tick organs by a blood feeding or pathogen injection. We have tested the ability of synthetic peptides def1 and def2 to inhibit the growth or directly kill several pathogens. The antimicrobial activities (expressed as minimal inhibition concentration and minimal bactericidal concentration values) against Gram positive bacteria were confirmed, while Gram negative bacteria, yeast, Tick Borne Encephalitis and West Nile Viruses were shown to be insensitive. In addition to antimicrobial activities, the hemolysis effect of def1 and def2 on human erythrocytes was also established.

**Conclusions:**

Although there is nothing known about the realistic concentration of defensins in *I. ricinus *tick body, these results suggest that defensins play an important role in defence against different pathogens. Moreover this is a first report of a one amino acid substitution in a defensins molecule and its impact on antimicrobial activity.

## Background

Ticks are known as vectors of severe human and animal diseases caused by viruses, bacteria and protozoa. Their immune system offers effective mechanisms against pathogenic microorganisms in the event of their permeation into the tick body. Some pathogen species (*Babesia *sp., *Borrelia *sp., Tick Borne Encephalitis Virus, etc.) can survive and colonize tick tissues and to be further transmitted to animal or human hosts during feeding [1].

The immune system of ticks and other arthropods possesses two sets of immune responses. The first one includes cellular responses represented by hemocytes and including encapsulation, nodulation and phagocytosis [2,3]. Hemocytes were shown to be one of the sites of immune molecule synthesis that play a role in humoral immune pathways [4]. The second set of immune responses in ticks consists of humoral responses, which involve the proteins that represent molecular factors of self/non-self recognition as well as effector molecules.

Defensins, naturally occurring antimicrobial peptides (AMPs), form the first line of defense against pathogens and have been found in a broad spectrum of living organisms: plants [5], mammals [6], insects [7-9], scorpions [10], mollusks [11] and several tick species [12-21]. Invertebrate defensins were first isolated from cultured cells of the flesh fly *Sarcophaga peregrina *[7]. Their amino acid sequence shows conservations across a broad phylogenetic range (ticks, insects, mollusks, and scorpions) suggesting these AMPs are relatively ancient effectors of innate immunity. Arthropod defensins are generally cationic AMPs having six cysteine residues forming disulfide bridges with the same pairing Cys1-Cys4, Cys2-Cys5, and Cys3-Cys6 [22]. In ticks, defensins are mainly expressed in the midgut after blood feeding or pathogen invasion [14,23,24]. Their antimicrobial activity is primarily directed against Gram-positive bacteria, but some isoforms are also effective against Gram-negative bacteria, yeasts and protozoa [24-27]. The initial interaction of defensins with bacterial cytoplasmic membranes involves electrostatic forces followed by permeabilization of the membranes and subsequently leading to bacterial lysis. It is thought that there may also be other, secondary, potential targets inside bacteria cells for cationic AMPs, such as nucleic acids or some enzymes [25,28].

Recently, multidrug resistant (MDR) bacterial strains have emerged and have rapidly spread in the environment, therefore the discovery of a novel class of antibiotics is urgently needed. During the course of evolution, nature has generated many molecules with conserved motifs that may represent specific probes for design of the new therapeutic agents. Characterization of these structures is of particular interest in order to produce molecules by chemical synthesis or recombinant systems, which may be candidates for new drug development [29]. Membrane-active cationic AMPs, like defensins, have the potential to become a new class of antibiotics with a new mode of interaction and promising therapeutical effects. These peptides are not yet affected by antibiotic-resistance mechanisms and, with various possible targets, the development of resistance might be difficult [28].

## Results

### Antimicrobial and hemolytic activities of the synthetic defensin isoforms

In the preliminary agar diffusion test, both synthetic isoforms of *Ixodes ricinus *defensin def1 and def2 revealed an antimicrobial activity against the Gram-positive bacteria *Staphyloccocus xylosus*, *Micrococcus luteus*, *Bacillus subtilis *and a clinical isolate of MDR strain of *S. aureus *(data not shown), but did not show any effect on Gram-negative bacteria (*Escherichia coli*, *Pseudomonas aeruginosa*) and yeast (*Candida albicans*). Subsequently the minimal inhibition concentrations (MICs) and the minimal microbicidal concentrations (MMCs) for selected sensitive bacteria species were determined. Synthetic def1 and def2 had an ability to inhibit Gram-positive bacteria in very low concentrations-specifically *M. luteus *(MIC 0.75 μM and 0.37 μM, respectively), *B. subtilis *(1.5 μM and 0.75 μM, respectively) and a methicilin resistant clinical isolate of *S. aureus *- MRSA (50 μM, and 25 μM, respectively). The minimal bactericidal concentrations were measured as the ability to kill 99.9% of bacteria in the culture. Both peptides showed the ability to kill tested bacteria within one hour, thought the MMCs of both peptides were slightly higher than the MICs. The values are summarized in Table 1.

**Table 1:** Minimal inhibition concentrations (MICs) and minimal microbicidal concentrations (MMCs) of def1 and def2 isoforms.

Bacterial, yeast and viral species	MIC [μM]		MMC [μM]	
	
	def1	def2	def1	def2
*Bacillus subtilis *(G+)	1.5	0.75	2	1

*Micrococcus luteus *(G+)	0.75	0.37	10	5

*Staphylococcus luteus *(G+)	50	25	50	25

*Escherichia coli *(G-)	no effect^a^	no effect^a^		

*Pseudomonas aeruginosa *(G-)	no effect^b^	no effect^b^		

*Candida albicans *(yeast)	no effect^b^	no effect^b^		

TBEV (Flaviviridae)	no effect^b^	no effect^b^	no effect^b^	no effect^b^

WNV (Flaviviridae)	no effect^b^	no effect^b^	no effect^b^	no effect^b^

Determination of MICs and MMCs for each peptide and bacteria was performed at least 3-times in doublets. Def2 peptide was significantly more efficient in the inhibition of growing G-positive bacteria (G+) in cultures and in microbicidal experiments. Gram negative bacteria (G-), as well as yeast, WNV and two strains of TBEV (Hypr and Neudoerfl), were not influenced by these peptides (^a ^no effect up to 50 μM; ^b ^no effect up to 100 μM).

Def1 as well as def2 isofoms exhibited no virucidal effect on the Tick Borne Encephalitis virus (TBEV), and West Nile virus (WNV). Moreover, no significant inhibition of TBEV, and WNV growth in a culture of PS cells was seen in the presence of the compounds. The only decrease (approx. 1 log_10 _pfu/ml) in WNV titer was seen in PS cell culture at 24 hours post-infection (p.i.) in the presence of def2 when compared to untreated control. However, no difference in virus titer between cultures treated with def2 and untreated controls was seen at 48 hours p.i. (data not shown). In conclusion, *I. ricinus *defensin isoforms did not reveal any substantial antiviral activity against TBEV, as a representative of tick-transmitted viral pathogens, and WNV, as a representative of mosquito-borne viruses.

**Figure 1:**
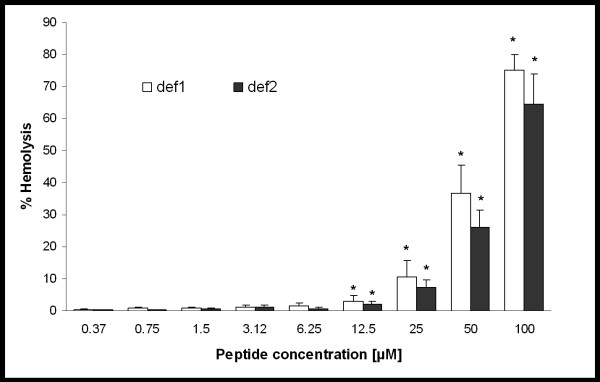
**Hemolytic effect of def1 and def2**. Hemolytic activity was carried out using 2% (vol/vol) erythrocyte suspension to evaluate all mild impacts on the erythrocyte membrane [40]. Normally, the 4-5% (vol/vol) erythrocyte suspension is used for the hemolysis experiments [41,42]. Significant differences between negative controls and peptides are marked by an asterisk (p < 0.05). No differences between hemolysis by def1 and def2 were statistically determined.

To determine the effects of *I. ricinus *defensins on mammalian cells, we measured the level of hemolysis caused by def1 and def2 in concentrations effective at killing Gram-positive bacteria. The results show that both peptides are harmless to human erythrocytes in concentrations of up to 12.5 μM (p < 0.05). In these concentrations def1 and def2 caused 2.9% and 2.0% hemolysis, respectively. The hemolysis rose to 75% and 64.6% respectively, when the highest concentrations of def1 and def2 were used (Figure 1).

The amino acid sequences of def1 and def2 differ only in one amino acid residue in position 8, where phenylalanine exists in case of def1 and arginine in case of def2. This substitution results in slightly different antimicrobial potential of def1 and def2. Def2 isoform is more effective in the inhibition of bacteria cell growth and killing of bacteria than def1 peptide. However, no significant difference was noticed in the degree of erythrocyte hemolysis.

### Stage- and tissue-specific expression profiles of *I. ricinus *defensin isoforms

**Figure 2:**
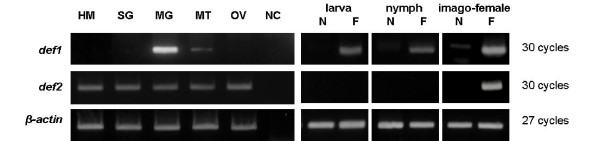
**Life cycle and tissue gene expression of *I. ricinus def1 *and *def2 *isoforms**. Semi-quantitative two steps RT-PCR was performed with specific primers for each defensin. Tissues for examination were obtained from blood fed female and were as follows: HM-hemocytes, SG-salivary glands, MG - midguts, MT - Malphigian tubes, OV - ovaries. Negative control was done without cDNA. Life stages of *I. ricinus*tick used were larvae, nymphs and adult females (N-unfed, F-fed). Gene for tick *β-actin*was used as a positive control.

**Figure 3:**
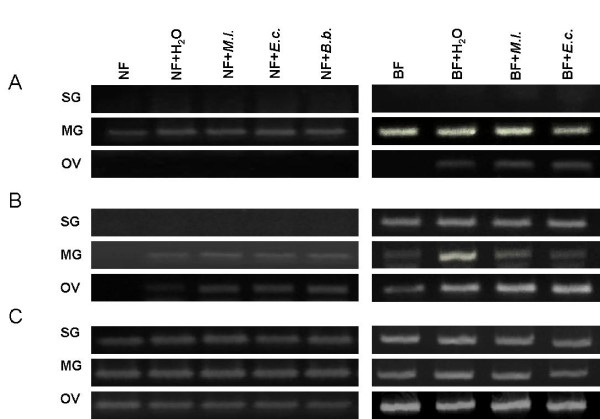
**Expression patterns of *I. ricinus *defensins isoforms *def1 *(A) and *def2 *(B) after various stimuli in comparison to house-keeping gene *actin *(C)**. Before (NF) or after a completed blood feeding (BF), ticks were inoculated intrahemocoelly with a suspension of bacteria (*E. coli*, *M. luteus*, O.D._600 _= 0.2; *Borrelia burgdorferi*sensu stricto, 4.8 × 10**^7^**spirochetes/ml; volume 0.5 μl). As a negative control one group of ticks was injected with the same volume of sterile water. RNA from all groups of ticks was isolated the next day. Expression of genes for *def1*(A) and *def2*(B) were determined by semi-quantitative two steps RT-PCR (30 cycles for BF ticks, 33 cycles for NF ticks) in salivary glands (SG), midgut (MG) and ovaries (OV). Gene for tick *β-actin*(C) was used as a positive control (27 cycles).

Semi-quantitative two step RT-PCR was performed to determine defensin gene expression in all developmental stages and in selected tissues before and after blood feeding or hemocel injection of different substances. Expression of *def1 *mRNA was induced by blood intake at all life stages - larva, nymph and imago, while *def2 *mRNA expression was detected only in adult females with a significant increase after a blood meal (Figure 2). *Def1 *was expressed in mitgut in both cases-before and after blood meal, while its expression in Malphigian tubes and ovaries was induce after blood intake or injection of different compounds into blood fed female, respectively (see Figure 2, 3a). Results of tissue specific expression patterns analysis for *I. ricinus def2 *isoform in response to blood feeding, injection of bacteria or sterile water (Figure 2, 3a,) showed that expression of *def2 *isoform was present in midgut and ovaries of unfed female after injection and blood feeding was a big stimulus for *def2 *expression in all tested organs (Figure 2, 3b) House-keeping gene, *β-actin*, was expressed at the same expression levels in all samples (Figure 2, 3c).

Newly designed primers for amplification of *I. ricinus β-actin *gene were confirmed as specific not only for *I. ricinus*, but also for the *I. scapularis *and *Dermacentor marginatus β-actin *gene. The obtained products were sequenced and partial sequences of *β-actin *gene of *I. ricinus *and *D. marginatus *were submitted to GeneBank (accession no. HQ682101; HQ645110, respectively).

## Discussion

### Functional characterization of def1 and def2

Molecules involved in the mechanism of innate immunity of invertebrates are a rich source of potential novel candidates for antibiotic development. Antimicrobial proteins (AMPs) - mainly defensins, play an important role in invertebrate immunity pathways. Recently there has been greater attention paid to this protein family. Here, we present the functional characterization of *Ixodes ricinus *defensins (def1, def2), previously described in our lab [18,19]. Defensins are known to be effective mainly against Gram-positive bacteria [24,25,27]. Our results confirmed this statement showing that *I. ricinus *defensin isoforms possess an effective antimicrobial activity against Gram positive bacteria *S. xylosus*, *M. luteus*, *B. subtilis *and MRSA clinical isolate *S. aureus*. Def1 and def2 isoforms show a strong ability to inhibit Gram-positive bacteria growth in low doses (see Table 1). Furthermore, def1 and def2 antimicrobials have the advantage of being bactericidal. Within one hour, 99.9% of tested bacteria were killed and observed MMCs corresponded to MICs, showing high efficiency of *I. ricinus *defensins. Neither def1 nor def2 affected the growth of tested Gram-negative bacteria (*E. coli *and a multi-resistant clinical isolate of *P. aeruginosa*) and yeast *C. albicans *in tested concentrations (Tab. 1). Generally, Gram-negative bacteria are more resistant to treatment of cationic antimicrobial peptides. Their cell wall, mainly the outer membrane, is known to be an effective permeability barrier [27,30]. It is possible that this is the reason the peptides cannot directly interact with the cell membrane. Despite the fact that the majority of findings showed tick defensins as mainly anti Gram-positive bacteria peptides, longicin, the defensin of the Asian tick *Haemaphysalis longicornis*, was also able to inhibit Gram-negative bacteria (*P. aeruginosa*, *E. coli*), a fungus (*Pichia pastoris*) and merozoites of an intracellular parasite *Babesia equi *[26]. Weak antibacterial activity of *I. persulcatus *defensin was observed against the Gram-negative bacterium *E. coli *[24].

Bacteria naturally co-existing with the tick, such as the symbiotic bacterium *Stenotrophomonas maltophila *(from the midgut of the tick *I. persulcatus*), the pathogenic spirochete *Borrelia garinii *(*Ixodes *ticks) or *I. persulactus*- derived *Bacillus *sp., revealed the natural resistance to tick defensins [27,31]. Here we have demonstrated that TBEV (virus derived from *I. ricinus *tick) and its life cycle were not influenced by tick defensins def1 and def2. Lyme disease spirochetes (*B. garinii*), classified as Gram-negative bacteria, were not sensitive to defensins synthesized according to the sequences derived from vector (*Ixodes ricinus*, def1) and non-vector ticks *Haemaphysalis longicornis *or *Ornithodoros moubata *(defC). [27].

Although the antiviral activity of several defensins (human, murine) was reported, the *I. ricinus *defensins did not substantively influence WNV and its life cycle in addition to TBEV [32,33].

Therapeutic alternatives for the treatment of multi-drug resistant bacteria infections are restricted to antibiotics introduced recently to clinical practice [34]. Recently, many human pathogens have emerged with multi resistance to known antibiotics [35]. Therefore, the basic antimicrobial testing should be a part of all research with probable or expected antimicrobial molecules. As was mentioned earlier, *I. ricinus *def1 and def2 showed antibacterial activity against G-positive bacteria. A methicilin resistant clinical isolate of *S. aureus *(MRSA) was sensitive to these peptides as well. Even if the MIC and the MMCs values of def1 and def2 peptides against *S. aureus *(MRSA) were higher than in other tested Gram-positive bacteria, this finding showed the ability of defensins to inhibit and kill pathogenic bacterium with multi-drug resistance. In contrast, *S. aureus *strain Wood was more sensitive (MIC_def1 _40 μg/ml; MW 4231.9; equivalent 9.45 μM concentration) than the MRSA strain to *Ixodes ricinus *def1 peptide treatment [27].

These findings, supplemented by the fact that these defensins have relative low toxicity to human cells (measured by the degree of red cell hemolysis; Figure 1), suggest that *I. ricinus *tick defensins (mainly def2 isoform) are possible candidates for new antimicrobial (especially anti-MRSA species) drug development with potential topical application.

### Impact of defensin primary structure on its function

The importance of forming the intra-molecular disulphide bonds between six cysteine residues in the defensin molecule had been reported before and was summarized by Ganz and Lehrer [22]. Specific S-S formation (between Cys1 - Cys4, Cys2 - Cys5 and Cys3 - Cys6) is vital for defensin function. The linear synthetic defensin from *I. persulcatus *displayed weak antibacterial activity in the growth inhibition test in comparison to the folded peptide with a confirmed tertiary structure of the same defensin peptide [31]. Nevertheless, Tsuji and colleagues [26] showed that the most important part of defensin molecule responsible for antimicrobial activity is the sequence of the C-terminal 20 amino acids. This shorter peptide (signed as C4 peptide) exerts the same antibacterial activity as a full length and folded recombinant mature defensin.

**Figure 4:**
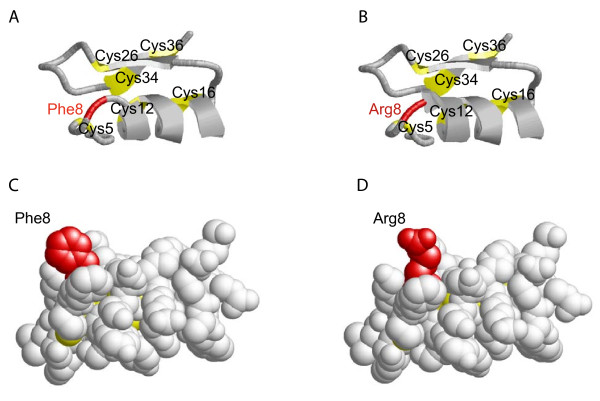
**Predicted 3D structures of def1 (A) and def2 (B) peptides and the impact of amino acid substitution on molecular surface (C, D)**. The predicted 3D structures (A, B) show no significant differences, but the amino acids residues of Phe8 (def1) and Arg8 (def2) projected out of the molecule surface (C, D; respectively). Because the arginine carries out the positive charge, the impact on molecule def2 cationicity is without doubt. Seeing that the defensins mode of action is a thorough depolarization of the bacteria cytoplasm membrane [25], the cationicity of peptides could be the explanation for def2 higher efficiency in killing bacteria. The predicted tertiary structures of def1 and def2 peptides were based on the template 1fjnA - 3D structure of defensin MGD-1 from the mollusk *Mytilus galloprovincialis*[43]. Sequence similarity between MGD-1 and def1, def2 were 48.65% in both cases.

Here we have shown that a single amino acid substitution on the N-terminal of defensin molecule also influences the function of the peptide. Def1 and def2 isoforms differ in position 8 of the amino acid sequences. The substitution affects the ability to kill previously mentioned bacteria: def2 (Arg8) possesses greater ability to inhibit bacterial growth and kill bacteria than def1 isoform (Phe8). While the substitution doesn't affect the predicted tertiary structure of both peptides (Figure 4a, b), the peptide surface has changed: the amino acid residues of Phe8 and Arg8 are present on the surface (see Figure 4c, d). The two peptides also differ in cationicity - def 2 has higher predicted positive charge (7.052; at pH 7.0) and could probably be more efficient in bacteria cell membrane depolarization than isoform def1 (6.052; at pH 7.0).

### Defensins expression patterns

During feeding, ticks are naturally exposed to host blood environment possibly containing infectious agents. Activation of the tick innate immune responses is necessary for a successful defense against invading pathogens, and consequently for completing blood intake and the life cycle. Defensins, as a part of humoral immunity pathway, are induced by many stimuli. They are mainly up-regulated in the midgut by blood feeding [13,14,24,26]. It is speculative if defensins may play a role in blood digestion - our results show that def1 as well as def2 are able to slightly lyse erythrocytes up to 12.5 μM concentration. In our opinion a more probable explanation for the up-regulation of defensin gene expression after blood intake is the activation of the immune system due to history events of simultaneously intake of pathogens and blood meal. The results of gene expressions show defensins as the first line defense molecules in tick immunity. Gene for *def1 *was predominantly expressed in the midgut, while *def2 *was detected in all tested organs after different treatments. Our observations reveal that the immune system of *I. ricinus *females (measured by defensin expression) reacts on different pathogens almost equally. Analogously, there were reports of up-regulation of defensins gene expressions after a bacterial treatment in several other ticks. In the soft tick, *Ornithodoros moubata*, four defensin isoforms were induced by injection of different bacteria or bacteria cell wall components. These *O. moubata *defensin isoforms react slightly different to treatment of Gram positive or Gram negative bacteria [25]. Infection of the hard tick *D. variabilis *by Gram-negative bacteria *Anaplasma marginale *also leads to increasing defensin gene mRNA levels [23].

## Conclusions

In conclusion, *I. ricinus *defensins (def1, def2) play an important role in the tick's innate immunity. Their expression is mainly induced by a blood feeding - the most important process for pathogen entry. Therefore, the expression of both defensin isoforms is predominantly found in the midgut but sometimes in other organs such as salivary glands or ovaries as well. Both *I. ricinus *defensin isoforms possess bactericidal properties with potency predominantly against Gram-positive bacteria; the def2 isoform was found to be more effective in killing bacteria with the same level of human erythrocyte hemolysis as the def1 isoform. Generally, with the increasing emergence of multi-resistant bacteria, tick defensins as well as other arthropod defensins can be expected to be introduced to the medical field as new molecules with antibacterial activity.

## Methods

### Tick samples

*I. ricinus *ticks were provided by the Biological Centre, Institute of Parasitology, Academy of Science of the Czech Republic. Uninfected ticks of different life stages (larva, nymph and imago) were fed on adult guinea pigs (infection-free animals treated in accordance with the Animal Protection Law of the Czech Republic no. 246/1992 Sb). Ticks were collected after completing blood feeding (self-separation from the animal).

### Semi-quantitative two step RT-PCR analysis

Semi-quantitative two-step reverse transcriptase-polymerase chain reaction (RT-PCR) analysis was performed to analyze the gene expression of *Ixodes ricinus *defensins. Total RNA was extracted from all tick samples using the TRI reagent (Sigma, USA) according to the manufacturers' recommendations. RNA samples were obtained from unfed and fed larvae, nymphs and adult females and from dissected tissues unfed and fed females (hemolymph, midgut, salivary glands, ovary, and Malphigian tubes).

Single strand cDNA was prepared from the total RNA with random primers (0.2 μg per reaction) using RevertAid H Minus First Strand cDNA Synthesis Kit (MBI Fermentas, Lithuenia). Five micrograms of total RNA was used per each reaction. Synthesis of the first strand cDNA was carried out at 42°C for 60 minutes according to the manufacturers' recommendations.

Primers were designed according to *I. ricinus *defensin isoforms sequences previously obtained in our laboratory [18,19]. The mature defensins isoforms (*def 1*, *def 2*) were amplified using specific primers: Def1ma forward 5'- GGT GGC TAC TAC TGC CCA TTT TTT-3', Def2ma forward 5'- GGT GGT TAC TAC TGC CCA TTC CG-3'. The reverse primer (DefRma) sequence (5'- TCA GAC GCA GAT GCA GGT CTT TT-3') was the same for both defensin isoforms. The tick housekeeping gene encoding *β-actin *was used as a control in RT-PCR using the gen-specific primers; the primers were designed according to *Dermacentor variabilis *(EF488512) and *Rhipicephalus (Boophilus) microplus *(AY255624) actin sequences and were as follows (forward primer 5'-ATG TGT GAC GAC GAG GTT GCC GC-3', reverse primer 5'-GTA CAG CGA CAG CAC GGC CTG G-3'). To amplify the target gene, the single strand cDNA (150 ng per reaction) was used. Amplification was performed with GoTaq Colorless Master Mix (Promega, USA) in 20 μl. PCR conditions were as follows: 5 min at 96°C, followed by 28 cycles at 96°C for 25 s, 53°C (defensins) or 50°C (actin) for 30 s and 72°C for 1 min. Final extension step was at 72°C for 5 min. PCR was performed in a Mastercycler (Eppendorf, Germany) thermal cycler. Products of the reaction were separated by electrophoresis on 1.8% agarose gel electrophoresis.

*Def1 *and *def*2 PCR products, as well as the *β-actin *gene PCR product, were cut off the gel and purified using the QIAquick Gel Extraction Kit (Qiagen, USA) and subsequently cloned into pCR^®^4-TOPO vector (Invitrogen, USA) for sequencing to proof defensin isoform primers specificity. DNA sequencing was performed using ABI 3130 Sequencer and the Big Dye^® ^Terminator v3.1 Cycle Sequencing Kit (Applied Biosystems, USA) with M13 forward/reverse primers. Sequences were analyzed with DNASTAR software (DNASTAR, United Kingdom). BLAST programs of the National Centre for Biotechnology Information (Bethesda, USA) were used for similarity search using the data available in GenBank^®^.

### Preparation of synthetic tick defensins

Peptides def1 and def2 (mature parts, both 37 amino acid long) were custom synthesized according to putative amino acid sequences (accession numbers AAP94724 and ABC88432, respectively) by the Peptide 2.0 Company (Chantilly, VA, USA). Purification by reversed-phase high-performance liquid chromatography and MS analyses were performed by the vendor. According to the data reports supplied with the products, synthetic Def1 (GGYYCPFFQDKCHRHCRSFGRKAGYCGGFLKKTCICV) was 93.84% pure and its molecular weight was 4.237 kDa, while synthetic Def2 (GGYYCPFRQDKCHRHCRSFGRKAGYCGGFLKKTCICV) was 85.82% pure and its molecular weight was 4.247 kDa. The products were supplied as lyophilized trifluoroacetate salt which was stored at -76°C. Before experiments, the peptides were dissolved in PBS buffer containing 0.05% Tween 20 (Loba Feinchemie, Austria) and 1 μM *β*-merkaptoethanol (Bio-Rad, CA, USA) to final 1 mM concentration.

### Biological activity assays

Following organisms were used for determination of basic antimicrobial activity profile: *Bacillus subtilis *(*B. s*.) 168, kindly provided by Prof. Yoshikawa (Princeton University, Princeton, NJ, USA); *Escherichia coli *(*E. c*.) B No. CCM 7372 and *Micrococcus luteus *(*M. l*.) No. CCM 144 from the Czech Collection of Microorganisms, Brno; *Staphylococcus aureus *(*S. a*.) and *Pseudomonas aeruginosa *(*P. a*.) were obtained as multi-resistant clinical isolates, No. 4231 and 8567, respectively, from Liberec Hospital, Czech Republic; and *Borrelia burgdorferi *(*B.b*.) No. ATCC 35211 from American Type Culture Collection, Manassas, VA, USA. Yeast, *Candida albicans *F7-39/IDE99, kindly provided by the Institute of Organic Chemistry and Biochemistry, was a clinical strain from a collection of fungi at the Institute of Microbiology, Faculty of Medicine and Dentistry, Palacký University Olomouc, Czech Republic. For antiviral activity assay, two representatives of TBEV (strains Neudoerfl, and Hypr) and one isolate of WNV were used. The strain Neudoerfl was originally isolated from *I. ricinus *tick in Austria in 1971 [36], and the strain Hypr was isolated from the blood of a 10-year-old child with tick-borne encephalitis in 1953 [37]. Low-passage TBEV strains were used in this study. WNV isolate used in this study is of unknown origin and underwent 28 passages in brains of suckling mice.

#### Antimicrobial assay

Qualitative estimates of the antimicrobial activities of both synthesized *I. ricinus *defensin isoforms were performed by the agar dilution double-layer technique. One hundred micro litters of fresh bacterial culture (approximately 10^7 ^colony forming units per Petri dish) prepared in LB broth were added to 2 ml of melted soft agar (0.5% LB agar) once temperature decreased to approximately 42°C. Subsequently, soft agar was poured over the surface of the Petri dish containing 20 ml of 2% LB agar. Synthetic defensins 1 and 2 were diluted to concentration of 100 μM in physiological buffer and one microliter of either peptide was dropped on the surface of the solidified upper layer containing bacteria. As a negative control, dissolving buffer (PBS containing 0.05% Tween 20 and 1 μM *β*-merkaptoethanol) was used. Plates were incubated at 37°C overnight.

#### Minimal inhibition concentratins (MICs)

Quantitatively, MICs were established by observing bacteria growth in multi-well plates. Stock solutions (1 mM) of def1 and def2 were diluted in LB broth and put into the wells of multi well plates. Bacteria in mid-exponential phase were diluted (1 × 10^5 ^CFU/ml) and added to the wells with peptides (0.1 ml of diluted peptide and 0.1 ml of bacteria culture), final peptide concentration ranged from 0 to 100 μM. Diluted dissolving buffer in LB broth was used as a negative control. Multi-well plates were incubated in a Bioscreen C instrument (Helsinki, Finland) for 20 h (or 24 h for *M. luteus*) at 37°C, with constant shaking. The absorbance was measured at 540 nm every 15 min, and each peptide was tested in 3 independent experiments.

#### Minimal micorbicidal concentrations (MMCs)

The overnight bacterial cultures of *M. luteus*, *B. subtilis *and *S. aureus *were subcultured in fresh LB media at 37°C with shaking to obtain log-phase bacterial cells. Subsequently, bacteria cells were diluted to 1-2 × 10^6 ^cells/ml in PBS (pH 7.4). One hundred microlitres of cell suspension were mixed with 100 μl of peptide def1 or def2 solutions (0 - 200 μM) and incubated 1 hour without shaking at 37°C. Afterward the bacteria samples were diluted either 100 or 1000 times, spread on LB agar plates and incubated overnight at 37°C. Grown colonies were counted, CFU per ml and percentage of dead bacteria relative to controls was calculated. MMCs were evaluated as the lowest peptide concentration able to kill 99.9% of bacteria in 3 independent experiments.

#### Antiviral activity

Virucidal effect of defensin 1 and 2 on TBEV was investigated by incubation of 10^5 ^pfu of the virus in L15 medium (3% FCS) in the presence or absence of the def1 and def2 compounds at final concentration of 100 μM for 1 hour at 37°C. The residual infectivity was determined by a plaque assay as described previously [38].

The inhibitory effect of def1 and def2 on TBEV replication was investigated on PS cell monolayers (porcine kidney stable) [39]. The cells in 96-well plates were infected with 10^5 ^pfu per well in L15 medium (3% FCS). After one hour of adsorption at 37°C, the inoculum was removed by washing the cells with PBS. Subsequently, the cells were incubated in L15 medium in the presence or absence of the peptides. The concentration of defensins used in the experiment was 100 μM. Supernatant media from the wells were collected at 24 and 48 hours post-infection and stored at -70°C until processing. Virus titer was determined by the plaque assay as described previously [38].

#### Hemolytic assay

EDTA-anticoagulated human venous blood was obtained from 2 young healthy volunteers. Erythrocytes were harvested by centrifugation (2000 rpm, 10 min, and 20°C) and washed three times with sterile PBS. Suspension of erythrocytes (2%; vol/vol) was used for the assay. Stock solution of peptides def 1 and 2 were diluted in PBS and co-incubated with erythrocytes (37°C, 2 hours) in the final volume of 200 μl and final concentrations from 0.37 μM to 100 μM. After incubation, the suspension was centrifuged (2000 rpm, 10 min, and 20°C), 100 μl of supernatant was removed, and the absorbance of samples was measured at 405 nm (A_N_). The hemolytic activity was calculated in correlation to negative and positive controls (%hemolysis = (A_N _- A_0 _/A_100 _- A_0_) × 100; A_0 _= 0% hemolysis in PBS; A_100 _= 100% hemolysis obtained by incubation with 0.2% solution of Triton X-100 in PBS).

### Statistical analysis

The significance of any differences obtained between def1 and def2 peptides, concentrations used and controls was evaluated by the Mann-Whitney U test, non-parametrical (Statistica 6.0, Statsoft, Inc. Tulsa, USA). The results represent 5 similar doublet experiments for hemolytic activity and 3 doublet tests for antimicrobial activity.

### Tertiary structure prediction

For prediction of the 3-D structure of both def1 and def2 peptides, the Swiss-Model Protein Modeling Server was used [http://swissmodel.expasy.org/SWISS-MODEL.html] to find the homologues for *I. ricinus *molecules. A pdb file of the molecule (according to the similar sequences of structures from the protein databank) was downloaded and used in RasWin Molecular Graphics program (Version 2.7.5; http://www.rasmol.org/) to view the 3D structure of both peptides.

## Competing interests

The authors declare that they have no competing interests.

## Authors' contributions

TC carried out molecular biology work, functional analysis, and statistics; participated in MIC establishment and antiviral tests and drafted the manuscript. JS carried out MIC establishment. DR carried out the antiviral activity tests. LG and NR participated in the study design and helped to draft the manuscript. All authors read and approved the final manuscript.
